# Phospholipid Scramblase 1 Modulates FcR-Mediated Phagocytosis in Differentiated Macrophages

**DOI:** 10.1371/journal.pone.0145617

**Published:** 2016-01-08

**Authors:** Cecile Herate, Ghania Ramdani, Nancy J. Grant, Sabrina Marion, Stephane Gasman, Florence Niedergang, Serge Benichou, Jerome Bouchet

**Affiliations:** 1 Inserm U1016, Institut Cochin, Paris, France; 2 CNRS, UMR8104, Paris, France; 3 Université Paris-Descartes, Sorbonne Paris-Cité, Paris, France; 4 Institut des Neurosciences Cellulaires et Intégratives, CNRS UPR3212, and Université de Strasbourg, Strasbourg, France; Institut Curie, FRANCE

## Abstract

Phospholipid Scramblase 1 (PLSCR1) was initially characterized as a type II transmembrane protein involved in bilayer movements of phospholipids across the plasma membrane leading to the cell surface exposure of phosphatidylserine, but other cellular functions have been ascribed to this protein in signaling processes and in the nucleus. In the present study, expression and functions of PLSCR1 were explored in specialized phagocytic cells of the monocyte/macrophage lineage. The expression of PLSCR1 was found to be markedly increased in monocyte-derived macrophages compared to undifferentiated primary monocytes. Surprisingly, this 3-fold increase in PLSCR1 expression correlated with an apparent modification in the membrane topology of the protein at the cell surface of differentiated macrophages. While depletion of PLSCR1 in the monocytic THP-1 cell-line with specific shRNA did not inhibit the constitutive cell surface exposure of phosphatidylserine observed in differentiated macrophages, a net increase in the FcR-mediated phagocytic activity was measured in PLSCR1-depleted THP-1 cells and in bone marrow-derived macrophages from PLSCR1 knock-out mice. Reciprocally, phagocytosis was down-regulated in cells overexpressing PLSCR1. Since endogenous PLSCR1 was recruited both in phagocytic cups and in phagosomes, our results reveal a specific role for induced PLSCR1 expression in the modulation of the phagocytic process in differentiated macrophages.

## Introduction

Phospholipid scramblase 1 (PLSCR1) is a member of a protein family referenced as phospholipid scramblases that are conserved in all eukaryotic organisms. In human, the scramblase family is constituted of four known homologues named PLSCR1, 2, 3 and 4 [1]. As the most studied member of the scramblase family, the 37 kD ubiquitous PLSCR1 protein has been described as a type-II transmembrane protein comprised of a short 9 amino acid (aa)-long C-terminal extracellular domain (aa 310–318), a single transmembrane helix (aa 291–309) and a long intracytoplasmic N-terminal domain of 290 aa (aa 1–290), containing a cysteine-rich palmitoylation motif (C_184_CCPCC_189_) that could stabilize PLSCR1 anchoring in biological membranes [2–4]. PLSCR1 mutants with substitutions in this palmitoylation motif have been shown to localize in the nucleus where PLSCR1 can also carry out biological functions, such as transcriptional activity [5].

The main function ascribed to PLSCR1 has been related to its potential involvement in bidirectional and nonspecific movements of phospholipids between the inner and outer leaflets of the plasma membrane in response to intracellular calcium mobilization [6–8]. Scrambling of membrane phospholipids then leads to the cell surface exposure of phosphatidylserine (PS), a critical signal for biological processes such as cell activation, coagulation, apoptosis and secretion [9,10]. However, this specific role of PLSCR1 in regulating phospholipid movements within the plasma membrane has been recently challenged in several experimental systems (for reviews, [2,9]).

While the exact involvement of PLSCR1 in the translocation of membrane phospholipids remains controversial, increasing evidence now indicates that this transmembrane protein could also be involved in cell signaling processes at the plasma membrane. Indeed, PLSCR1 is found in lipid rafts where it has been shown to interact directly with several plasma membrane receptors, including the epidermal growth factor receptor, the high-affinity IgE receptor FcɛRI and the CD4 T-cell receptor [11–14]. In T lymphocytes, we have shown that both PLSCR1 and PLSCR4 are cellular receptors for the secretory leucocyte protease inhibitor (SLPI) and interact with CD4 at the plasma membrane [14]. In addition, PLSCR1 can also associate with cellular tyrosine kinases containing Src-homology 3 (SH3) domains, such as c-Abl [15] and Syk [16], and Src family kinases including Src and Lyn [13,16]. Association of PLSCR1 with these kinases is probably related to the multiple SH3-binding proline-rich motifs found in the long cytoplasmic domain of PLSCR1 (for review, [2]). However, the exact contributions of these interactions to specific functions of PLSCR1 are still poorly understood.

To further characterize these functions, PLSCR1 expression was first examined in CD4-positive myeloid and lymphoid cells, and PLSCR1 levels were found to be higher in monocytic cells than in T lymphocytes. We next analyzed the expression and potential functions of PLSCR1 in the professional phagocytic myeloid cells, monocytes and macrophages. We found that the level of PLSCR1 was markedly increased during differentiation of primary monocytes to macrophages, and more interestingly, PLSCR1 specifically modulated phagocytosis in differentiated macrophages.

## Materials and Methods

### Cell culture and differentiation

Adherent HeLa cells were grown in Dulbecco minimal essential medium supplemented with 10% fetal calf serum (FCS), 100 IU of penicillin/ml, and 100 μg of streptomycin/ml (Invitrogen). Human THP-1 monocytic and HPB-ALL T lymphoid cells have been already described [17]. THP-1 and HPB-ALL non-adherent cells were cultured in RPMI 1640 medium with Glutamax-1 (Invitrogen) supplemented with 10 mM HEPES, 10% FCS, 100 IU of penicillin/ml, and 0.1 mg streptomycin/ml (complete medium). For differentiation in macrophages, THP-1 cells were treated in complete medium, supplemented with 1 μM phorbol 12-myristate 13-acetate (PMA) (Sigma) alone or in combination with ionomycin where indicated, for the indicated time periods. Peripheral blood mononuclear cells (PBMCs) were isolated by Ficoll density gradient centrifugation from whole blood donated by healthy volunteers (Etablissement Français du Sang, Hôpital Saint Vincent de Paul, Paris, France). The study has been approved by the Ethic Committee from Inserm (Institut National de la Santé et de la Recherche Médicale, Paris, France), and subjects gave written, informed consent. PBMCs were incubated for 1 hour in a humidified atmosphere at 37°C with 5% CO_2_, and then washed twice in RPMI to keep only adherent cells. Monocytes were then differentiated into macrophages for 7 days in RPMI 1640 medium supplemented with 10% of human serum from the same donor. PLSCR-1 -/- mice were purchased from CDTA (Cryo-preservation, Distribution, Typage et Archivage animal), housed and raised at Chronobiotron UMS 3415. All mice were bred, handled, and maintained in agreement with European council directive 86/609/EEC and resulting French regulations. Mouse management and sample collection were conducted by trained personnel under the supervision of a veterinarian, in accordance with protocols approved by the "Institut National de la Santé et de la Recherche Médicale’s" ethical committee of animal welfare. Mouse bone marrow-derived macrophages (BMDMs) were isolated and cultured as previously described [18].

### Western blot analysis

Primary cells, THP-1 and HPB-ALL cells were resuspended in solubilization buffer containing 1% NP-40 (Sigma), 0.1 M (NH_4_)_2_SO_4_, 20 mM Tris (pH 7.5), 10% glycerol, and 1× protease inhibitor (Roche). After 30 min under gentle agitation, cell lysates were centrifuged at 14,000 × *g* for 30 min. The soluble fraction was then assayed for protein content with the DC protein assay kit (Bio-Rad), and 20 μg of proteins was incubated for 5 min at 95°C in Laemmli sample buffer containing 5% of β-mercaptoethanol. Proteins were resolved by sodium dodecyl sulfate-polyacrylamide gel electrophoresis on 12% acrylamide NuPAGE Novex bis-Tris precast gels (Invitrogen). PLSCR1 and ɣ-tubulin (control) were detected by Western blotting using the mouse 1E9 (Abcam) monoclonal antibody (mAb) directed against the PLSCR1 N-terminal domain and the anti-ɣ-tubulin (clone GTU-88, Sigma) mAb, respectively, followed by goat secondary HRP-coupled anti-mouse antibodies (Sigma). The intensity of the PLSCR1 band was quantified by densitometry using NIH Images Software as described previously [19]. Cell lysates of BMDM cultures were prepared and analyzed by Western blotting as previously described [20]. The absence of PLSCR1 expression in cultures from PLSCR1-/- mice was verified with a rabbit anti-PLSCR1 antibody raised against the N-terminus of murine PLSCR1 (4720 [14]; gift of Dr. P.J. Sims), and a monoclonal anti-β-actin antibody (Clone AC-15, Sigma) and a rabbit anti-Iba1 antibody (Wako Chemicals) were employed as controls.

### Flow cytometry analysis

For cell surface detection of PLSCR1 by flow cytometry, labeling was performed on ice on non-fixed cells to avoid permeabilization, and selection of non-permeabilized viable cell populations was assessed using propidium iodide (1 μg/ml) as described previously [17]. The PLSCR1 N-terminal domain was stained with the 1E9 mAb used at 4 μg/ml, while the PLSCR1 C-terminal domain was stained with a rabbit pAb [16] used at the 1:10 concentration as previously described [16]. PLSCR1 staining was then detected with anti-mouse Alexa488- and anti-rabbit Alexa647-coupled secondary antibodies (Molecular Probes), respectively. Staining of cell surface PS was performed using phycoerythrin-coupled AnnexinV (BD Pharmingen) for 15 min at room temperature as described previously [17]. For intracellular staining of PLSCR1, cells were fixed for 20 min in 4% formaldehyde and permeabilized using 0.1% Triton X-100 for 10 min at 4°C. Cells were then stained with the 1E9 mAb and Alexa488-coupled anti-mouse IgG. Cell surface CD14 was labeled using FITC-coupled mouse anti-CD14 (BD Pharmingen). All flow cytometry analyses were performed using a Cytomics® FC 500 cytometer, and data were analyzed with Cytomics RXP analysis software. The purity of BMDM cultures was evaluated on a non-fixed, viable cell population after blocking the Fc-receptor with rat anti-mouse CD16/32 (BD Pharmingen) and staining with the macrophage marker, V450-coupled rat anti-mouse CD11b antibodies (BD Pharmingen) using a MACSQuant Flow Cytometer.

### Immunofluorescence analysis

Non-treated THP-1 cells and primary monocytes were washed and resuspended in PBS and laid on coverslips pretreated with poly-L-lysine (0.1 mg/mL) (Sigma) for 20 min at room temperature. PMA-treated THP-1 and monocyte-derived macrophages (MDMs) were grown on non-treated coverslips, washed in PBS, and then fixed with 4% paraformaldehyde in PBS. Intracellular PLSCR1 was detected with the 1E9 mAb in 1% BSA-PBS, supplemented with 0.1% Triton X-100 for permeabilization, and then stained using Alexa488-coupled secondary antibody. Cell surface PLSCR1 was detected either with the 1E9 mAb or with the rabbit pAb. Samples were examined under an epifluorescence microscope (Leica DMB) with a cooled charge-coupled device camera (Micromax 1300Y/HS; Roper Princeton Instruments), using a Plan APO 100X objective. Images acquisition was perfomed with MetaMorph 7.6 (Molecular Devices). After culturing BMDMs for at least 10 days in 10 ng/ml mCSF (Miltenyi Biotec), cells were plated on non-treated coverslips, subsequently fixed and permeabilized as described above, and then labeled with TRITC-phalloidin (Sigma) and rabbit anti-Iba1 (Wako Chemicals GmbH) and an Alexa 647 coupled-goat anti-rabbit-IgG (Molecular Probes). Images were acquired using a confocal microscope (Leica SP5II) equipped with a Plan APO 63x objective (N.A. 1.40, pinhole 1.0).

### shRNA transduction

To stably knockdown PLSCR1 endogenous expression in THP-1 cells, two pLKO.1 lentiviral vectors harboring short hairpin RNAs (shRNA) targeting PLSCR1 (shPLSCR1) were obtained from Sigma. The 21-nucleotide sequences within *PLSCR1* targeted by the shRNA oligonucleotide pairs were 5’-CAGTTCCCTTTAGACCTTGAT-3’ and 5’-CCACCTGGATTAGAATATTTA-3’. First, vesicular stomatitis virus glycoprotein G (VSV-G)-pseudotyped lentiviral particles (LVPs) harboring shRNAs targeting PLSCR1 were produced separately in 293T cells by cotransfecting pLKO1-shRNA, an HIV-1 packaging vector (8.91, lacking the *env* and auxiliary genes), and a VSV-G expression plasmid as described previously [21]. The pLKO.1 plasmid expressing shRNA against firefly luciferase (shLuc) (Sigma) was used to produce control particles. At 48 h post-transfection, LVPs were pelleted from supernatants by ultracentrifugation (22,000 rpm for 1.5 h at 4°C), and amounts of CAp24 produced were determined by ELISA (Innogenetics) according to the manufacturer protocols. THP-1 cells (10^7^) were then transduced with 100 ng of CAp24 of either shLuc- or shPLSCR1-containing viral particles (50 ng of each shPLSCR1 LVP). 24 h later, transduced cells were cultured with fresh medium containing puromycin (1 μg/mL). After selection for 6 to 7 days, the level of PLSCR1 was evaluated by Western blotting and immunofluorescence using specific antibodies.

### Phagocytosis assay

For phagocytosis analysis in the THP-1 cell-line, cells were seeded and differentiated for 72 h on coverslips, at a density of 2x10^4^ cells in 1 ml of complete medium containing 0.5 μM PMA. Opsonization of sheep red blood cells (SRBCs) was performed extemporaneously as described previously [22]. SRBCs were washed twice in PBS-BSA 1% and then incubated with rabbit IgG anti-SRBCs at RT for 30 min (MP Biomedicals). After washing in PBS-BSA, opsonized SRBCs were resuspended in prewarmed phagocytosis medium (RPMI) and distributed on the cells grown on coverslips (SRBC:macrophage ratio = 10:1). Phagocytosis was first synchronized by centrifugation for 2 min at 400 g and then initiated by incubating cells in a humidified atmosphere at 37°C with 5% CO_2_. At 2, 5, 10 or 30 min, the coverslips were placed on ice, washed once with cold PBS, fixed in PBS containing 4% paraformaldehyde (PFA) and processed for immunofluorescence. Extracellular SRBCs were labeled with anti-Rabbit Alexa647-coupled IgG (Molecular Probes). Cells were then permeabilized in PBS-BSA containing 0.05% saponin during 30 min at RT, and labeled with the mouse anti-PLSCR1 1E9 mAb (Abcam) in PBS-BSA, 0.05% saponin. Intracellular staining was performed using anti-Rabbit Alexa350-coupled IgG for SRBCs, anti-mouse Alexa555-coupled IgG for PLSCR1 and phalloidine-Alexa 488 for F-actin (Invitrogen). To quantify phagocytosis, the number of internalized particles (SRBCs) was determined at 2, 5, 10 or 30 min in 50 randomly chosen cells, and a phagocytic index (i.e., the mean number of phagocytosed particles per cell) was calculated for each time point. The index obtained was expressed as the percentage of SRBCs phagocytosed after 30 min by shLuc-tranduced control cells. The number of cell-associated particles was also counted at each time point, to calculate the association index (mean number of external + internal particles per cell), which was then expressed as a percentage of control shLuc cells at 30 min. Samples were examined as described above. Z-series of images were taken at 0.2 μm increments. Deconvoluted images were obtained using Huygens Professional® software (Scientific Volume Imaging). Three-dimensional reconstructions were obtained using the IsoSurface function of Imaris software (version 7.4, Bitplane AG). For phagocytosis analysis in mouse BMDMs, cells were seeded on coverslips in complete RPMI medium complemented with 1 mM sodium pyruvate, 50 mM β-mercaptoethanol and 30% of L929 conditioned-medium. SRBCs were opsonised as described above, resuspended in prewarmed RPMI medium and distributed on the cells. Phagocytosis was synchronized by centrifugation, and uptake was initiated by incubating the cells at 37°C with 5% CO_2_. At 5, 10 or 30 min, phagocytosis was stopped by adding cold medium and placing cells were placed on ice, and samples were then processed for immunofluorescence. Before permeabilization, SRBCs were labeled with anti-Rabbit Alexa488-coupled IgG (Molecular Probes). Then, cells were fixed in PBS containing 4% PFA and intracellular staining was performed in PBS/BSA supplemented with 0.1% Triton X-100 (Sigma-Aldrich) using phalloidin-Alexa555 (Invitrogen) for F-actin and anti-Rabbit Alexa647-coupled IgG for SRBCs (Molecular Probes). Samples were examined as described above under an epifluorescence microscope (Leica DMB), and the number of internalized SRBCs in each cell was counted in at least 50 randomly chosen cells.

## Results

### Induction of PLSCR1 expression during monocyte-to-macrophage differentiation

**Fig 1:**
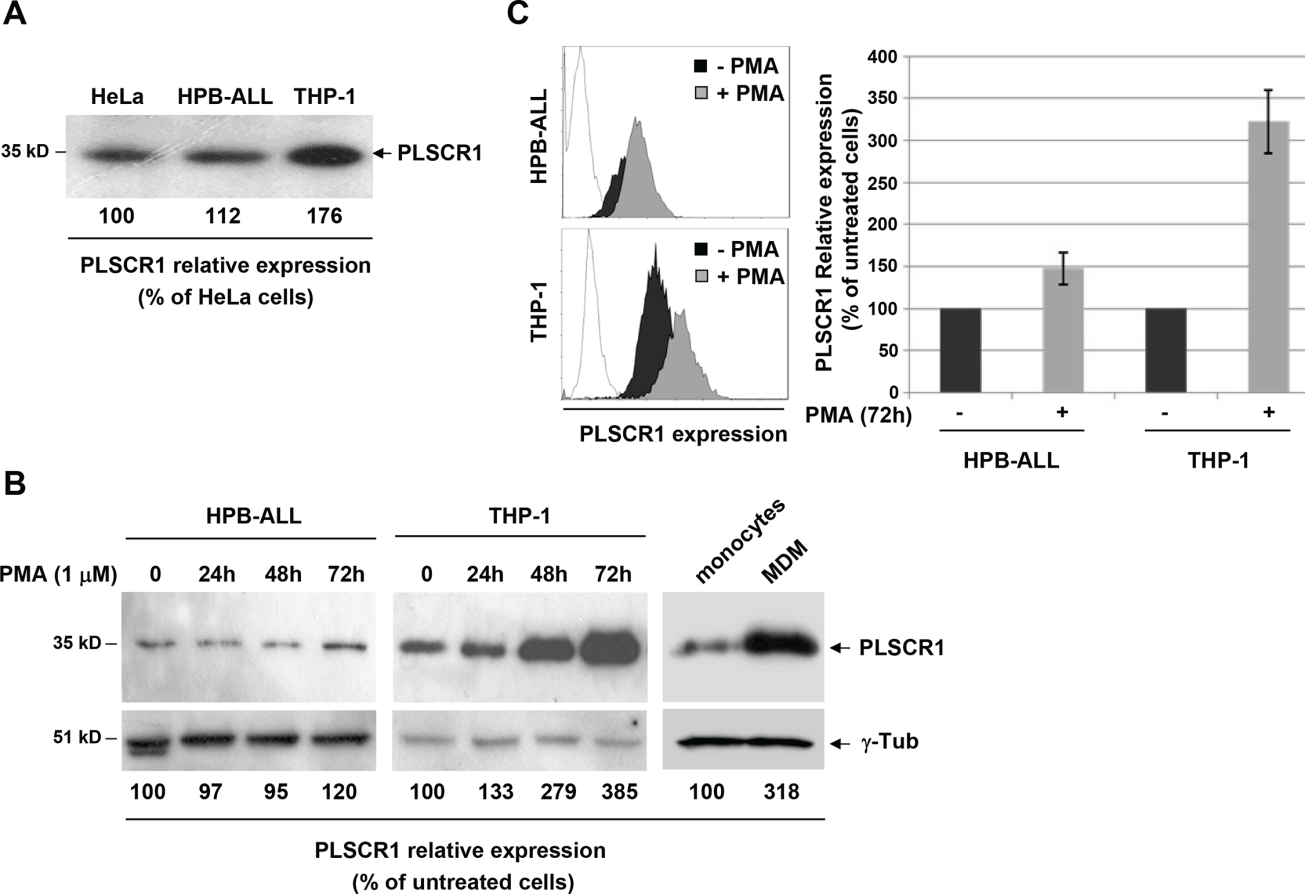
Intracellular expression of PLSCR1 in lymphoid and myeloid cells. (A) Equivalent amounts of total lysate protein from HeLa, HPB-ALL or THP-1 cells were resolved on SDS-PAGE and analyzed by Western blotting with anti-PLSCR1 antibody. The band intensities were quantified using NIH Image Software and the signals measured in HPB-ALL and THP-1 cells were normalized to the signal obtained for HeLa samples. The indicated values are expressed as percentages of the signal intensity relative to HeLa cells (100%), and are representative of three independent experiments. (B) HPB-ALL (left panels) and THP-1 (middle panels) cells were cultured in the presence of PMA-ionomycin and harvested at the indicated times, while primary monocytes were differentiated into macrophages (right panels) for 7 days in culture medium supplemented with the human serum from the same donor. Equivalent amounts of proteins from total cell lysates were analyzed by Western blotting with anti-PLSCR1 (upper panels) or anti-ɣ-tubulin (ɣ-Tub, lower panels). The values indicated represent the percentage of the signal intensity relative to non-treated cells or monocytes (100%). C) HPB-ALL and THP-1 cells were cultured for 72 h with or without PMA-ionomycin. Cells were permeabilized and stained with anti-PLSCR1 antibody followed with Alexa488-conjugated anti-mouse IgG, and intracellular expression of PLSCR1 was measured by flow cytometry. Representative experiments are shown on the left with dotted curves corresponding to permeabilized cells stained only with Alexa488-conjugated anti-mouse IgG. The means of 3 independent experiments are reported on the right with results expressed as the percentage of the mean fluorescence intensity (MFI) relative to untreated cells; error bars represent 1 standard deviation (SD) from the mean.

The expression level of endogenous PLSCR1 in lysates of human CD4-positive lymphoid and myeloid cells was first evaluated and compared with PLSCR1 expression in epithelial HeLa cells by Western blotting (Fig 1A). In monocytic (THP-1) and lymphocytic (HPB-ALL) cell-lines, as in HeLa cells, a single 35 kD band corresponding to PLSCR1 was detected in total cell lysates. However, the relative expression level of PLSCR1 was significantly higher in the promonocytic THP-1 cells, compared to either HeLa or HPB-ALL cell lines. Since this analysis was performed in resting cells, PLSCR1 expression was then examined in lysates from HPB-ALL T cells and THP-1 cells treated for different time periods with PMA-ionomycin. As shown in Fig 1B, the relative PLSCR1 expression was not significantly modified in lysates from treated HPB-ALL T cells, even after a PMA-ionomycin treatment for 72 h or longer (data not shown). In contrast, a net increase in PLSCR1 expression was already observed in THP-1 cells after a 24 h treatment, and attained a 3–4-fold increase by 72 h. Flow cytometry analysis of total PLSCR1 expression in permabilized HPB-ALL and THP-1 cells confirmed these results (Fig 1C). In addition, the 3-fold increase in PLSCR1 expression observed in treated THP-1 cells was found to be maintained in cells cultured for longer periods or in the presence of PMA alone (data not shown). Since the treated THP-1 cells had a morphologically differentiated macrophage phenotype and showed a decrease in the cell surface expression of CD14, a monocyte marker that is downregulated during differentiation [23,24], these data indicate that PLSCR1 is upregulated during PMA-induced differentiation of the promonocytic THP-1 cells. Finally, PLSCR1 expression was examined during differentiation of primary circulating monocytes in macrophages (MDMs). Primary monocytes were isolated from peripheral blood mononuclear cells (PBMCs) and then differentiated into macrophages. As shown in Fig 1B (right panels), a similar 3-fold induction of PLSCR1 expression was observed in macrophages compared to monocytes from the same donor. Together, these results demonstrate that expression of PLSCR1 is specifically upregulated during monocyte-to-macrophage differentiation.

### Apparent modification of the PLSCR1 membrane topology and cell surface exposure of phosphatidylserine during monocyte-to-macrophage differentiation

**Fig 2:**
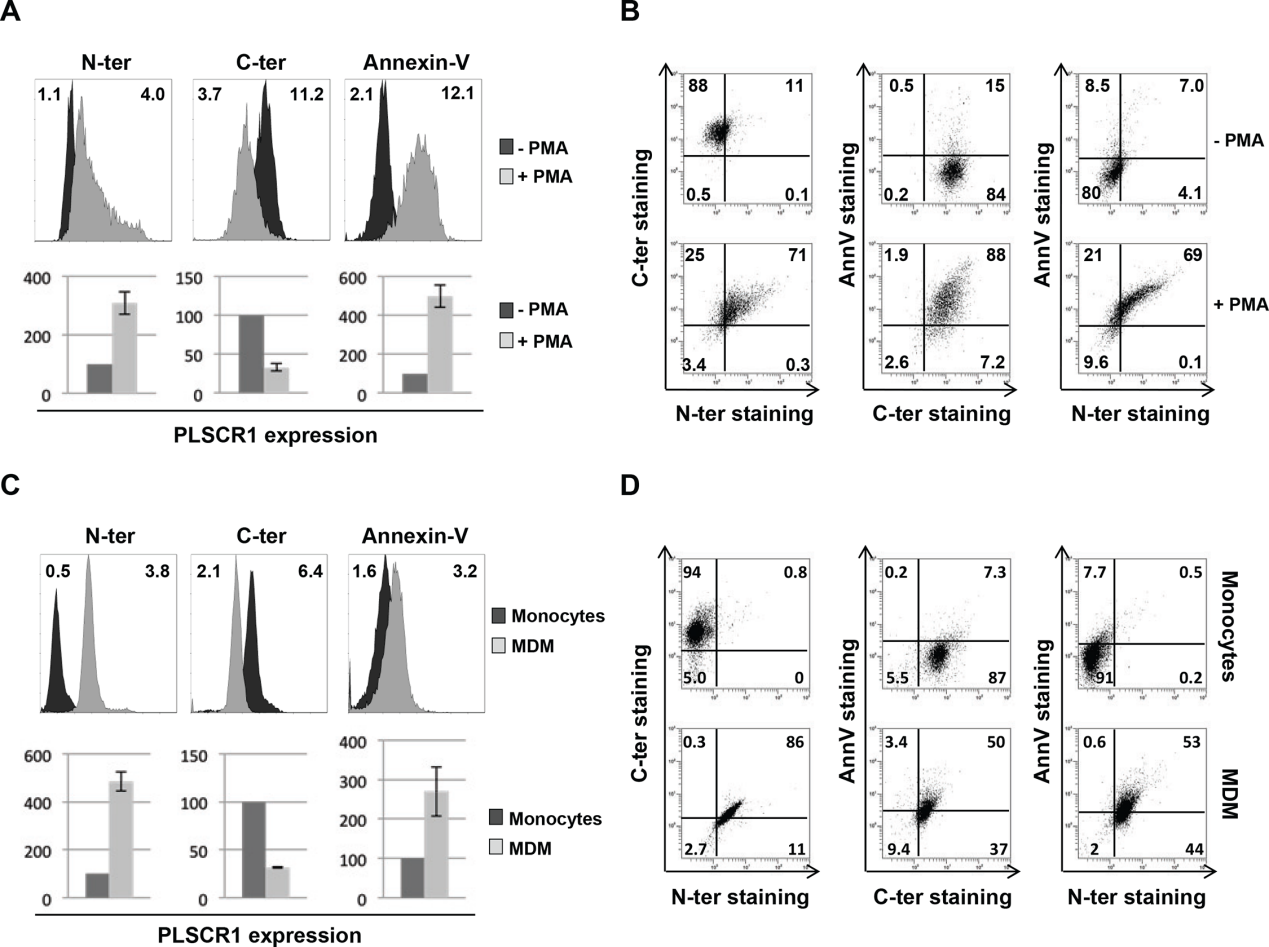
Cell surface expression of PLSCR1 during monocyte differentiation into macrophages. THP-1 cells (A and B) were cultured for 72 h in the presence or absence of PMA, while primary monocytes (C and D) were differentiated in macrophages as indicated in Fig 1. (A and C) Cell surface staining was performed at 4°C with anti-N-ter-PLSCR1 (1E9 mAb) followed by an Alexa488-anti-mouse IgG (left panels), anti-C-ter-PLSCR1 (rabbit pAb) followed by an Alexa555-anti-rabbit IgG (middle panels), or PE-conjugated Annexin-V (right panels). In upper panels of (A) and (C), MFIs are indicated on histograms, and in the lower panels, these results are quantified and expressed as a percentage of the mean value obtained for non-treated THP-1 cells (A) or monocytes (C). Values are the means of 3 independent experiments. Error bars represent 1 SD from the mean. (B and D) Representative biparametric analyses of the cell surface staining with anti-N-ter-PLSCR1 and anti-C-ter-PLSCR1 antibodies, and Annexin-V are shown for treated and untreated THP-1 cells (B), and monocytes and MDMs (D). In (B) and (D), the percentage of cells in each quadrant is indicated.

We then analyzed by flow cytometry whether the level of PLSCR1 expression at the plasma membrane of monocytes and macrophages correlated with cell surface exposure of PS. Since PLSCR1 is described as a type-II transmembrane protein, flow cytometry analyses were first performed on non-permeabilized cells, stained on ice with a polyclonal serum (pAb) directed against the short C-terminal extracellular domain of the protein, and a monoclonal antibody (mAb) that specifically recognizes an epitope located in the N-terminal domain of PLSCR1 was used as a control [16]. Using propidium iodide staining to assess cell permeability, the analysis of PLSCR1 staining was restricted to the propidium iodide-negative, non-permeabilized cell population. Unexpectedly, a specific surface staining was measured on non-permeabilized THP-1 cells with the anti-N-terminus mAb, and this staining was enhanced after 72 h of PMA-induced differentiation (Fig 2A, left panels, and Fig 2B). Conversely, staining with the anti-C-terminus pAb was decreased in PMA-treated cells compared to non-treated cells (Fig 2A, central panels, and Fig 2B), suggesting a change in the membrane topology of PLSCR1 molecules expressed at the plasma membrane after PMA-induced differentiation. Interestingly, these observations correlated with a 5-fold increase in PS exposure at the surface of differentiated THP-1 cells (Fig 2A, right panels, and Fig 2B, central and right panels). Similar results were obtained when primary monocytes were differentiated into macrophages with autologous human serum (Fig 2C and 2D). A 5-fold increase in PLSCR1 molecules with N-terminal epitopes exposed at the cell surface was measured together with PS exposure in macrophages, whereas a significant decrease in the cell surface presentation of the C-terminal domain of the protein was concomitantly observed during differentiation (Fig 2C and 2D). These results indicate that PLSCR1 overexpression and cell surface exposure of PS observed during monocyte-to-macrophage differentiation correlate with an apparent modification in the membrane topology of the protein at the cell surface of differentiated macrophages.

### Cell surface expression and intracellular distribution of PLSCR1 during monocyte-to-macrophage differentiation

**Fig 3:**
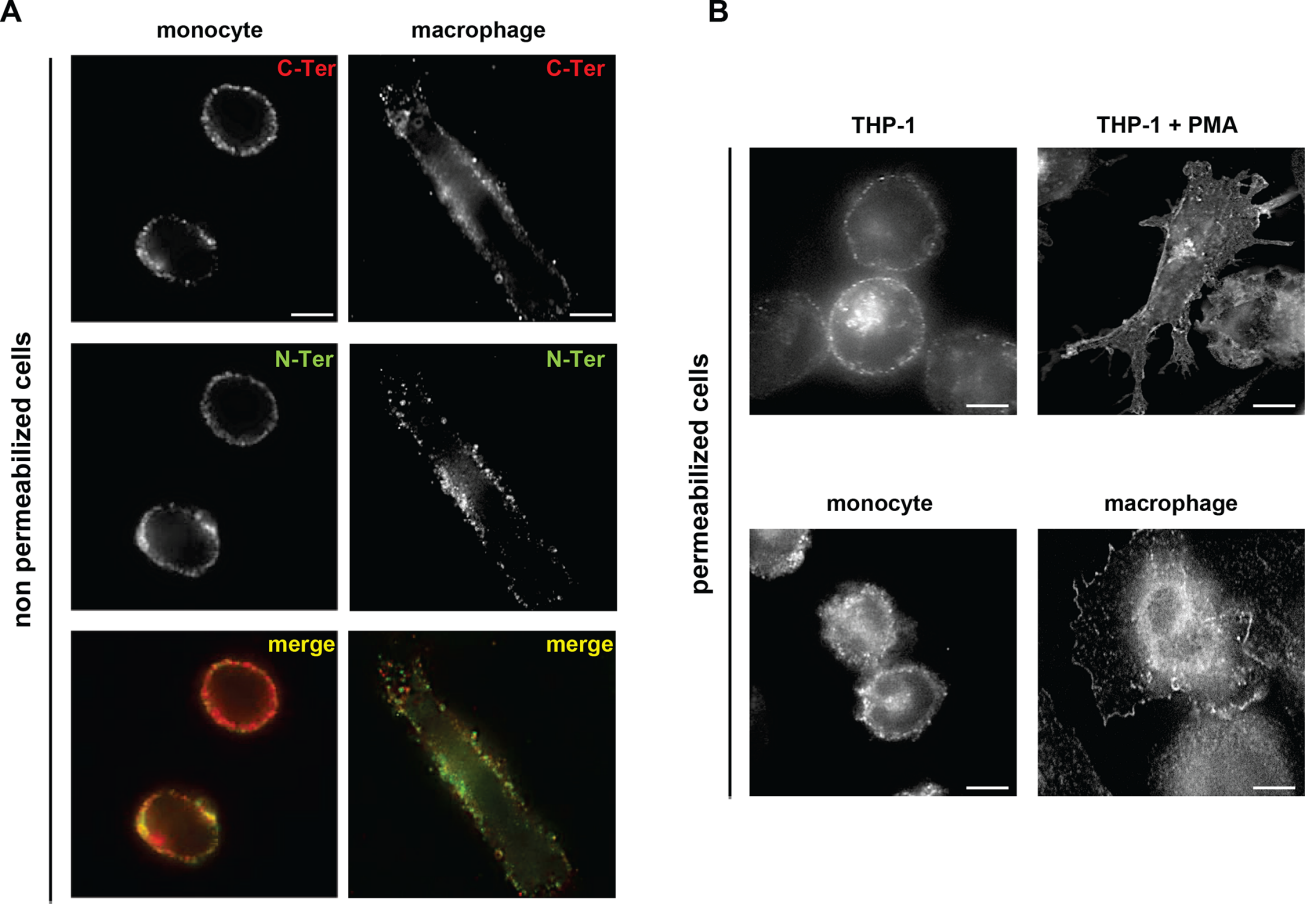
Cellular distribution of PLSCR1 during monocyte-to-macrophage differentiation. **(**A) Monocytes (left images) were differentiated into macrophages (right images) as described in Fig 1 and cell surface expression of PLSCR1 was detected by indirect immunofluorescence at 4°C using anti-C-ter-PLSCR1 (rabbit pAb) followed with Alexa555-anti-rabbit IgG (upper images), or anti-N-ter-PLSCR1 (1E9 mAb) followed with Alexa488-anti-mouse IgG (middle images). (B) THP-1 cells (upper images) were cultured for 72 h with or without PMA, while primary monocytes were differentiated into macrophages (lower images) as indicated previously. After fixation and permeabilization, PLSCR1 was detected by indirect immunofluorescence with anti-PLSCR1 (1E9 mAb). Scale bars, 10 μm.

Immunofluorescence analyses were then performed on non-permeabilized primary cells in order to analyze whether PLSCR1 molecules with distinct membrane orientations could be visualized at the cell surface of monocytes and macrophages (Fig 3A). Surface staining of PLSCR1 on primary monocytes and MDMs with the anti-C-terminus pAb revealed positive spots at the plasma membrane (upper panels), indicating that PLSCR1 molecules displaying the expected type-II membrane orientation are present at the cell surface in both non-differentiated and differentiated primary cells. When cells were stained with the anti-N-terminus mAb, a dotted weak staining of PLSCR1 was also observed on both monocytes and MDMs. As shown in Fig 3A, both C-terminal or N-terminal epitopes of PLSCR1 molecules could be detected at the cell surface in a single cell. While a significant number of N-terminus and C-terminus-stained spots seemed to co-localize at the plasma membrane, others were clearly located in different areas of the membrane.

The intracellular distribution of PLSCR1 was then analyzed in permeabilized primary monocytes and MDMs, and in PMA-treated and untreated THP-1 cells (Fig 3B). In primary monocytes and untreated THP-1 cells (left panels), PLSCR1 was mainly localized at the plasma membrane as dotted structures as well as in a perinuclear compartment. In differentiated MDMs and PMA-treated THP-1 cells (right panels), PLSCR1 exhibited a pronounced intracytoplasmic dotted staining with a concentration of the protein in the perinuclear region and a more thin and diffuse staining pattern at the plasma membrane. Together, these results indicate that during monocyte-to-macrophage differentiation, some epitopes of PLSCR1 which are normally in the cytoplasmic domain become exposed at the cell surface. This suggests a potential change in the membrane topology of PLSCR1 molecules at the plasma membrane in differentiated macrophages.

### PLSCR1 is not required for the constitutive phosphatidylserine exposure at the cell surface of differentiated macrophages

**Fig 4:**
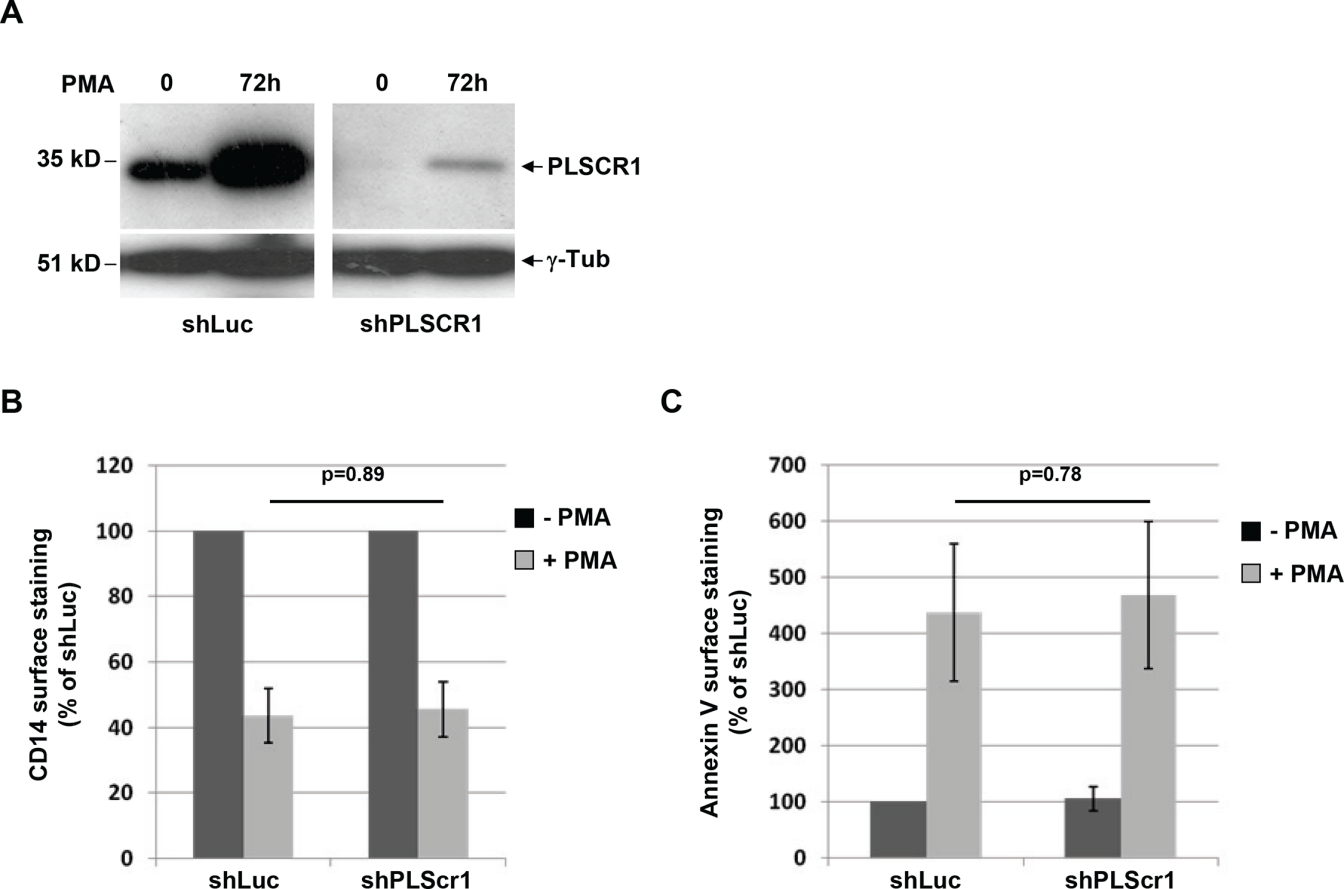
Monocyte-to-macrophage differentiation and cell surface phosphatidylserine exposure in PLSCR1-depleted cells. THP-1 cells were transduced with lentiviruses expressing shRNA against either PLSCR1 or Luciferase used as a control, and then cultured for 72 h with or without PMA as previously. (A) Lysates from shRNA-transduced THP-1 cells were analyzed by Western blotting with anti-PLSCR1 (upper panels) and anti-ɣ-tubulin (lower panels). (B and C) Cell surface expression of CD14 and PS exposure. Treated or untreated shRNA-transduced THP-1 cells were stained with FITC-conjugated anti-CD14 antibodies (B) or PE-conjugated Annexin-V (C), and surface expression was measured by flow cytometry. Results are expressed as the percentage of the MFI relative to the non differentiated control shLuc-transduced cells. Values are the means of 3 independent experiments. Error bars represent 1 SD from the mean. Statistical significance was determined using Student's *t* test (non significant, p > 0.05).

**Fig 5:**
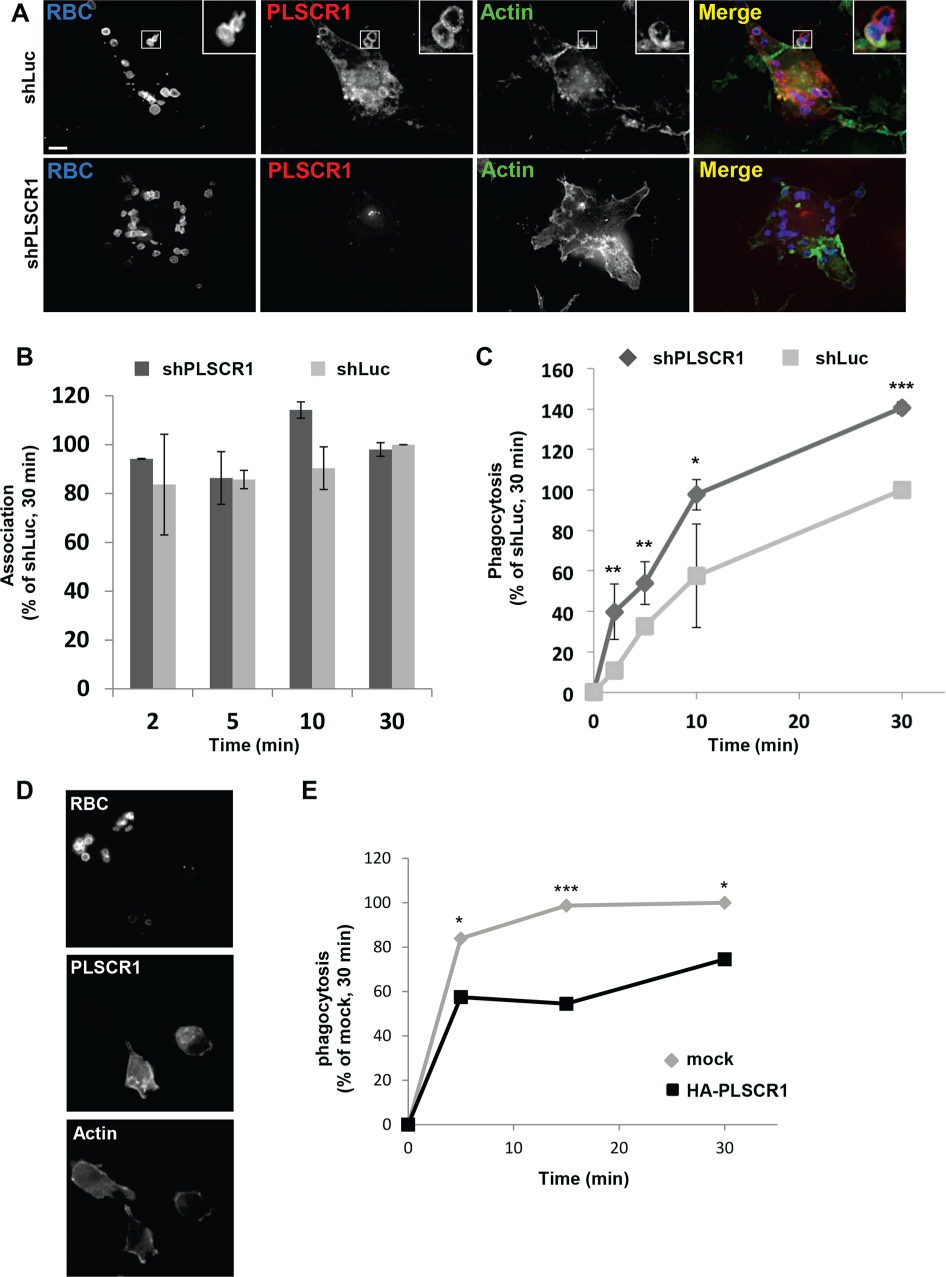
Phagocytosis activity in PLSCR1-depleted and -overexpressing cells. (A-C) THP-1 cells were transduced with lentiviruses expressing shRNA against PLSCR1 or Luciferase, treated with PMA for 72 h as indicated in Fig 4, and then submitted to phagocytosis of IgG-opsonised SRBCs. (A) shLuc- (upper panels) or shPLSCR1-transduced (lower panels) cells were incubated for 30 min at 37°C with opsonised SRBCs, then fixed, permeabilized and stained with Alexa350-anti-rabbit IgG, anti-PLSCR1 (1E9 mAb) revealed with Alexa555-anti-mouse IgG, and Alexa488-phalloidin to reveal internalized SRBCs, PLSCR1 and F-actin, respectively. (B and C) shLuc- or shPLSCR1-transduced cells were incubated for 2, 5, 10 or 30 min at 37°C with opsonised SRBCs, then fixed and stained with Alexa647-anti-rabbit IgG to detect external SRBCs before permeabilization for labeling of all cell-associated SRBCs including internalized particles with Alexa350-anti-rabbit IgG. The number of external SRBCs and the total number of cell-associated SRBCs in each cell was counted for at least 100 cells in each experiment. In (B), results are expressed as the percentage of cell-associated (internal and external) SRBCs/cell relative to that measured in shLuc-transduced cells after 30 min of phagocytosis. In (C), results are expressed as the percentage of phagocytosed SRBCs/cell relative to that measured after 30 min of phagocytosis in shLuc-transduced control cells. (D and E) THP-1 cells were transfected with the vector for expression of HA-PLSCR1, and 18 hours later tested for phagocytosis of IgG-opsonised SRBCs. In (D), transfected cells were incubated for 30 min at 37°C with opsonised SRBCs, then fixed, permeabilized and stained with Alexa647-anti-rabbit IgG, anti-HA rat antibody revealed with Alexa488-anti-rat IgG, and Alexa555-phalloidin to reveal internalized SRBCs, HA-PLSCR1 and F-actin, respectively. In (E), cells were incubated for 5, 15 or 30 min at 37°C with opsonised SRBCs, then fixed and stained with Alexa647-anti-rabbit IgG to detect external SRBCs before permeabilization for labeling of all cell-associated SRBCs including internalized particles with Alexa350-anti-Rabbit. Cells were also stained with anti-HA rat antibody revealed with Alexa488-anti-rat IgG to identify transfected cells. The number of external SRBCs and the total number of cell-associated SRBCs in each cells were counted for at least 50 transfected cells and 50 non-transfected cells. Results are expressed as the percentage of phagocytosed SRBCs/cell relative to that measured after 30 min of phagocytosis for non-transfected control cells. Values are the means of 3 independent experiments. Error bars represent 1 SD from the mean. Statistical significance was determined using students *t* test (n.s., p > 0.05; *, p < 0.05; **, p < 0.01; ***, p<0.001).

Because the main function of PLSCR1 was related to the movement of phospholipids between the inner and outer leaflets of the plasma membrane, we first investigated whether PLSCR1 expression induced during differentiation was required for the constitutive cell surface exposure of PS observed in macrophages compared to undifferentiated monocytes [25]. Lentiviruses were developed for expressing specific shRNAs targeting PLSCR1 and then used for depleting the protein in the monocytic THP-1 cell-line. Western blot analysis of cell lysates from transduced cells (shPLSCR1, Fig 4A) indicated that PLSCR1 was efficiently reduced in the lysates from shPLSCR1-transduced THP-1 cells, both before and after PMA-induced differentiation. In contrast, PLSCR1 expression was still enhanced in PMA-treated THP-1 cells transduced with control shRNA (shLuc, Fig 4A). Downregulation of endogenous PLSCR1 was confirmed in shPLSCR1-tranduced THP-1 cells by immunofluorescence, however, the PMA-treated PLSCR1-depleted cells still displayed a differentiated macrophage phenotype (see Fig 5A). In addition, flow cytometric analysis of PMA-treated and untreated cells revealed that the cell surface expression of the differentiation CD14 marker was downregulated in both shPLSCR1- and shLuc-transduced cells (Fig 4B), suggesting that the enhanced PLSCR1 expression is not essential for monocyte-to-macrophage differentiation. Finally, PS exposure at the cell surface was analyzed using flow cytometry after Annexin-V staining of PLSCR1-depleted THP-1 cells, before and after PMA-induced differentiation. As expected, a 4–5-fold increase of PS exposure was observed at the surface of shLuc control cells treated with PMA, compared to untreated shLuc cells. This increase was maintained on shPLSCR1-transduced THP-1 cells treated with PMA (Fig 4C). These results indicate that PLSCR1 expression is not required for the constitutive cell surface exposure of PS observed during monocyte-to-macrophage differentiation.

### Depletion of PLSCR1 stimulates phagocytosis in differentiated macrophages

Since macrophages are cells specialized in phagocytosis of microorganisms, apoptotic cells and red blood cells, we next explored whether PLSCR1 could be involved in this specialized function in PLSCR1-depleted cells. The phagocytic capacity of shLuc- and shPLSCR1-transduced THP-1 cells differentiated by PMA treatment was first tested by measuring FcR-mediated phagocytosis of IgG-opsonized sheep red blood cells (IgG-SRBCs). PMA-treated THP-1 cells were incubated at 37°C with IgG-SRBCs for 2, 5, 10 or 30 min, fixed and processed for immunofluorescent staining to detect external SRBCs before permeabilization, followed by the intracellular labeling of all cell-associated SRBCs, including internalized particles. Phagocytosis efficiency was quantified by calculating the index of phagocytosis at each time point, and the results are expressed as the percentage of SRBCs internalized by shLuc-transduced control cells after 30 min. As shown in Fig 5A, shPLSCR1-transduced cells ingested more IgG-SRBC particles than control shLuc-transduced cells after 30 min of incubation, indicating that phagocytosis was more efficient in PLSCR1-depleted cells. Although no significant difference was observed in the total number of IgG-SRBCs associated with PLSCR1-depleted cells compared to shLuc-transduced control cells (Fig 5B), PLSCR1-depleted cells displayed a significant increase in their phagocytic efficiency compared to control shLuc-transduced cells (Fig 5C). This increase was already apparent in shPLSCR1-transduced cells after 2 min with IgG-SRBCs, suggesting an acceleration of the internalization process in these cells. This difference in IgG-SRBCs internalization remained significant throughout the 30 min period of observation, showing that the depletion of endogenous expression of PLSCR1 stimulated phagocytosis by differentiated THP-1 cells. In agreement with this observation, phagocytosis was conversely found to be downregulated in transfected cells overexpressing ectopic HA-PLSCR1 (see Fig 5D and 5E). As shown in Fig 5D, less ingested IgG-SRBC particles were observed in cells overexpressing PLSCR1 compared to non-transfected cells after 30 min of incubation. The decrease in phagocytosis in PLSCR1 overexpressing cells was already apparent after 5 min and remained significant throughout the 30 min period of observation (Fig 5E).

**Fig 6:**
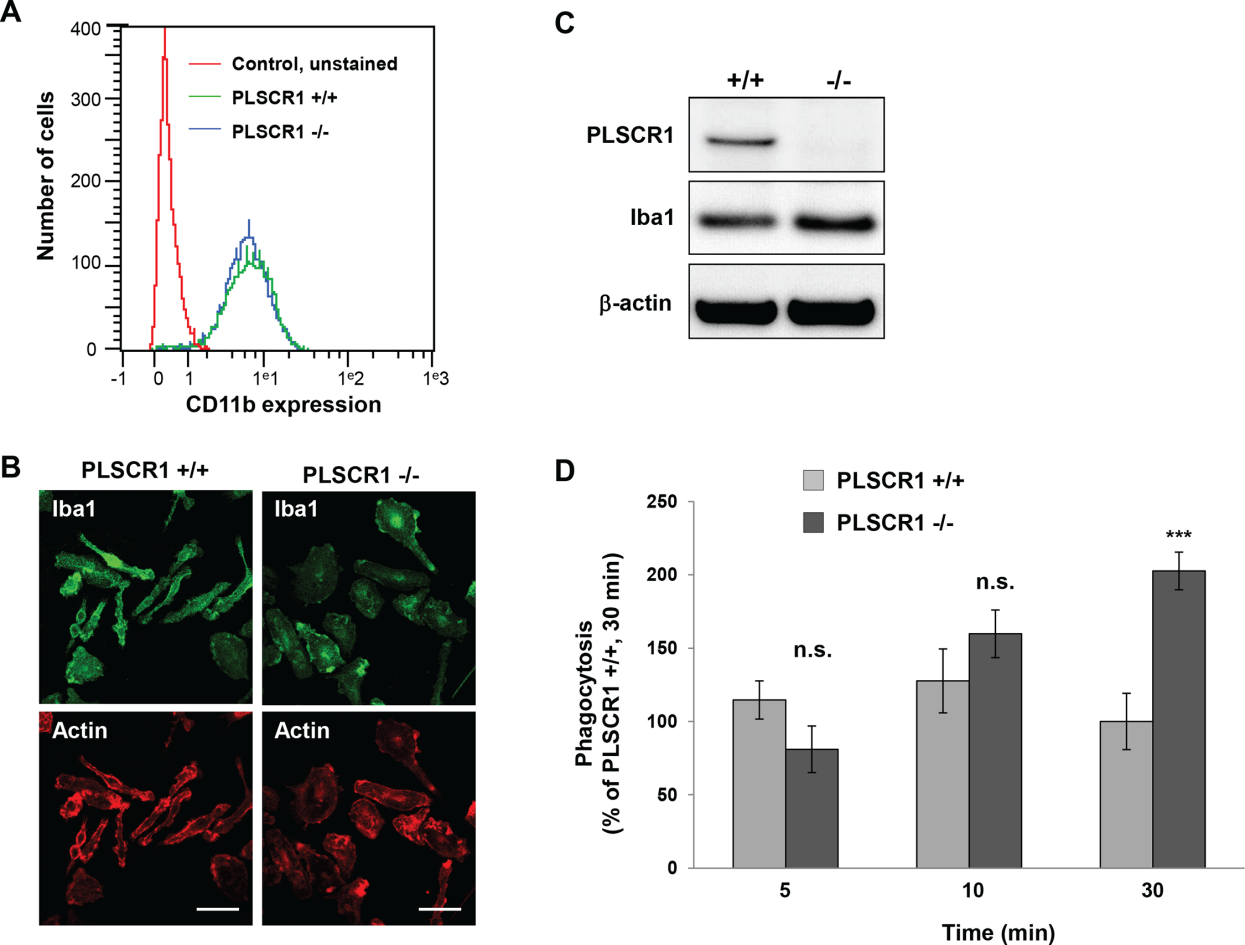
Characterization of bone marrow-derived macrophages from PLSCR1 kwock-out mice and their phagocytotic activity. **(**A) Histogram of CD11b expression by cells in BMDM cultures obtained from flow cytometric analysis of viable cells labelled with V450-anti-CD11b antibodies. Control sample was unstained. Nearly all cells (97%) in both PLSCR1 +/+ and PLSCR1 -/- cultures expressed the macrophage marker CD11b with similar MIFs, 6.7 and 7.0 respectively. (B) Confocal images of BMDM cultures prepared from PLSCR1 +/+ and PLSCR1 -/- mice. Cells were fixed and stained for F-actin with TRITC-phalloidin and macrophages were immunolabeled for Iba1 and visualized with a secondary antibody conjugated to Alexa 647. Bars, 50 μm. (C) Western blot of cell lysates (20 μg of total protein) of BMDM cultures from PLSCR1 +/+ and PLSCR1 -/- mice confirm the absence of PLSCR1 in knock-out mice. Blots were revealed with antibodies against PLSCR1, Iba1 and β-actin as loading control. (D) BMDMs from PLSCR1 +/+ (grey bars) or -/- (black bars) mice were incubated for 5, 10 or 30 min at 37°C with IgG-opsonized SRBCs, stained with Alexa488-anti-Rabbit IgG to detect external SRBCs before permeabilization and fixation for labeling of all associated SRBCs including internalized particles with Alexa647-anti-Rabbit IgG. The number of external SRBCs and the total number of cell-associated SRBCs were counted for at least 50 cells. Results are expressed as the percentage of cell-associated (internal and external) SRBCs/cell relative to that measured after 30 min of phagocytosis in BMDMs from PLSCR1 +/+ mice. Statistical significance was determined by the non parametric statistical hypothesis using the Mann-Whitney U test (n.s., p>0.05; ***, p<0.001).

To confirm the involvement of PLSCR1 in the regulation of the phagocytic function of macrophages, the phagocytic efficiency of bone marrow-derived macrophages from PLSCR1 knockout mice was examined. BMDMs purified from wild-type (PLSCR1 +/+) and knockout (PLSCR1 -/-) mice expressed similar levels of the macrophage markers CD11b (Fig 6A) and Iba-1 (Fig 6B). Western blot analysis confirmed the absence of PLSCR1 expression in BMDMs from knockout mice (Fig 6C). The phagocytic capacity of these BMDMs was tested by incubating them with IgG-SRBCs for 5, 10 or 30 min, and then processing them for immunofluorescent staining, as described above. The respective number of external and total cell-associated SRBCs in each cell was counted and the results are expressed as the percentage of SRBCs internalized by PLSCR1 +/+ control cells after 30 min (Fig 6D). While no significant differences were observed between PLSCR1 +/+ and -/- cells after 5 and 10 min, the number of SRBCs internalized by PLSCR1 -/- cells was significantly higher after 30 min of incubation. These results indicate that phagocytosis was more efficient in PLSCR1 -/- BMDMs than in wild-type cells, further confirming that PLSCR1 expression negatively regulates phagocytosis in differentiated macrophages.

### PLSCR1 is recruited to the phagocytic cup and phagosomes

**Fig 7:**
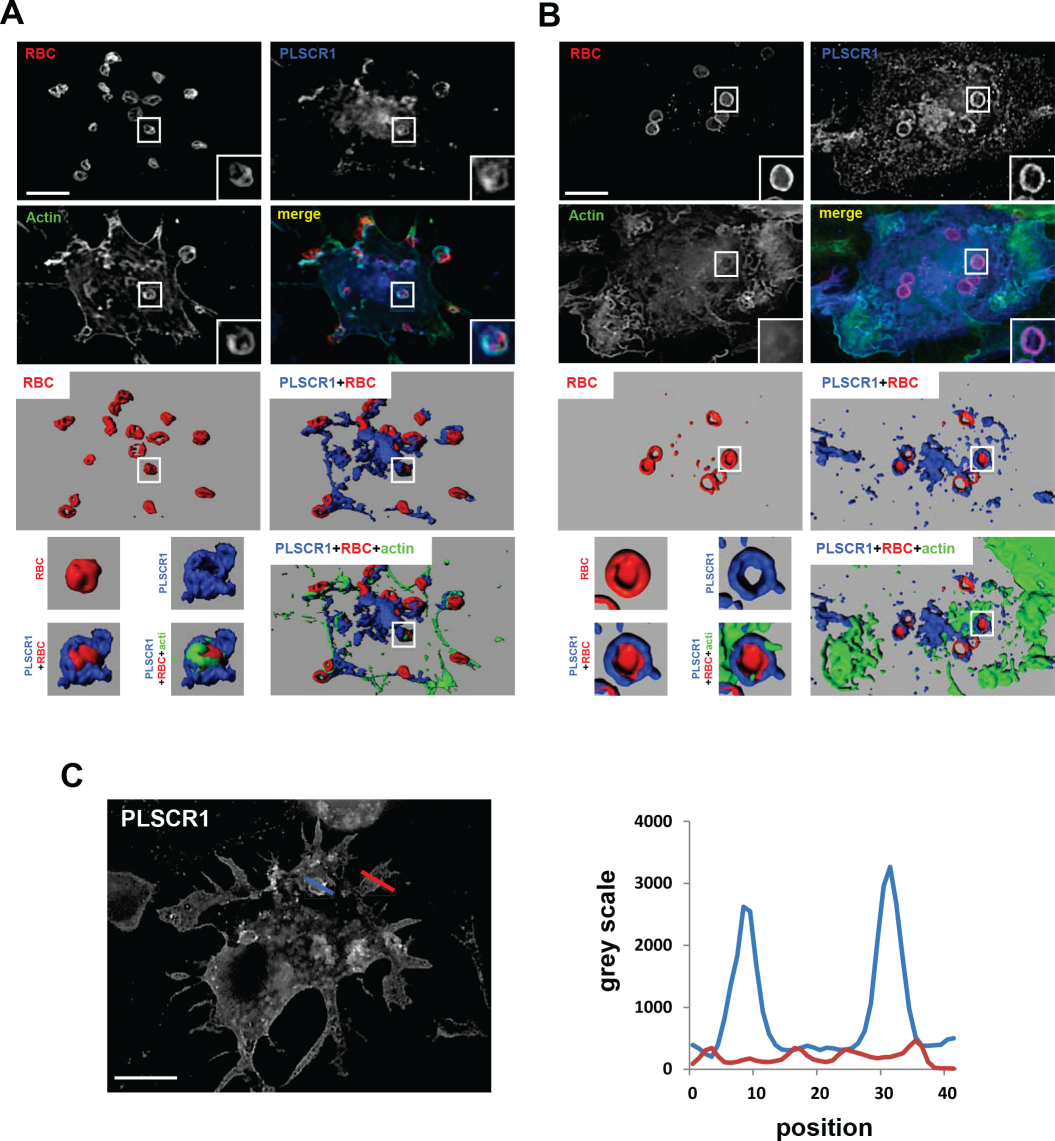
Recruitment of PLSCR1 at the phagocytic cup and on phagosomes. THP-1 cells were treated with PMA for 72 h as previously, and then incubated with IgG-opsonised SRBCs for 10 (A) and 30 (B) min at 37°C. Cells were then fixed and stained with Alexa647-anti-rabbit IgG to reveal external SRBCs. Internalized SRBCs in closed phagosomes, PLSCR1 and actin were stained after permeabilization with Alexa350-anti-rabbit IgG, anti-PLSCR1 1E9 followed with Alexa555-anti-mouse, and Alexa488-phalloidin, respectively. A and B) Representative images of phagocytic cups (A) and phagosomes (B). Z stacks of wide-field fluorescent images were acquired using a piezo, with an increment of 0.2 μm in the Z-axis, and the sum projection of 5 contiguous optical sections is shown (upper images). The stacks of images are presented as 3-dimensional reconstructions (middle panels), and left lower panels correspond to the enlargements of middle panels. (C) The profile of PLSCR1 fluorescence intensities (right panel) along the lines drawn at the phagocytic site (blue line) and the cell body (red line) (left image) are shown. Scale bars, 10 μm.

To gain further insight regarding the involvement of PLSCR1 in phagocytosis, the intracellular distribution of PLSCR1 in THP-1 cells was investigated during phagocytosis. Differentiated THP-1 cells were allowed to phagocyte opsonized IgG-SRBCs for 10 (Fig 7A) or 30 (Fig 7B) min and were then analyzed by fluorescence microscopy after staining for PLSCR1 together with F-actin. Whereas F-actin polymerization at sites of particle attachment is required in the early steps of the phagocytic process and defines a phagocytic cup, polymerized actin is quickly released from closed phagosomes (for review, [26]). As shown in Fig 7, PLSCR1 staining could be found in association with both F-actin-positive (Fig 7A, 10 min) and -negative (Fig 7B, 30 min) SRBC particles, suggesting that PLSCR1 is recruited to phagocytic cups and remains present on fully internalized phagosomes. Three-dimensional reconstitution of fluorescence images confirmed that PLSCR1 is specifically recruited at sites of phagocytosis, but does not colocalize with F-actin (Fig 7A). While actin was then released from closed phagosomes, PLSCR1 remained associated with internalized SRBC particles (Fig 7B). To quantify PLSCR1 recruitment on phagosomes, the fluorescence associated with engulfed particle was measured and compared with the signal obtained in other regions of the cell (Fig 7C). PLSCR1 was found to be enriched on phagosomes by a factor 3.27 +/-0.62 compared to the total fluorescence intensity in the cell, confirming the association of PLSCR1 with internalized phagosomes.

Together with the results of the phagocytosis assays, these data indicate that PLSCR1 participates in the modulation of phagocytosis in macrophages.

## Discussion

While PLSCR1 has been described as a protein widely expressed in most tissues, we show in the present study, using different experimental approaches, that PLSCR1 expression was strongly induced in human macrophages differentiated from primary monocytes. This increase in PLSCR1 expression was recapitulated in promyelocytic leukemic THP-1 cells after inducing differentiation into macrophage-like cells with phorbol esters, in agreement with previous reports for U937 and NB4 cells [27]. In contrast, no significant overexpression of PLSCR1 was observed in T lymphoid cell lines, HPB-ALL and CEM (data not shown) following PMA treatment. This net enhancement of PLSCR1 expression during differentiation of primary macrophages and monocytic cell-lines suggested that PLSCR1 might play a role in specific macrophage functions.

Surprisingly, induction of PLSCR1 expression in differentiated macrophages was accompanied by exposure of some epitopes from the N-terminal region of the protein on the cell surface, as evidenced by flow cytometry and immunofluorescence analyses. PLSCR1 is predicted to be a type-II transmembrane protein with a short C-terminal extracellular domain and a long N-terminal intracytoplasmic domain [6–8]. Our observations indicate a modification in the membrane topology of PLSCR1 molecules expressed during differentiation of monocytic transformed cell-lines and primary monocyte/macrophage cells. As expected, topology prediction software Toppred and TMpred (www.expasy.ch/tools/) indicate the presence of a single transmembrane helix spanning amino acids 291–309, with a preferential orientation of the short C-terminal domain on the cell surface (data not shown). While the actual membrane topology of PLSCR1 is still unknown, these programs predict an alternative PLSCR1 topology, whereby PLSCR1 adopts a type-I transmembrane orientation exposing the N-terminal region at the cell surface using the same transmembrane domain. Additional investigation is needed to determine whether the type-I topology of PLSCR1 corresponds to *de novo* synthesis of new molecules transported to the cell surface during differentiation or to mobilization of PLSCR1 proteins from the intracellular perinuclear membrane compartment (e.g., endoplasmic reticulum and Golgi apparatus) where PLSCR1 is concentrated both in monocytes and macrophages (see Fig 3).

Membrane protein topogenesis is governed primarily by a set of topogenic signals encoded in the amino acid sequence (for review, [28]), but recent evidence suggests that the membrane lipid composition is an important determinant in defining topology of transmembrane proteins [29]. Lipids have been implicated in the proper folding or changes in protein structure of some membrane-associated proteins in mammalian cells. For example, the ductin channel [30], a component of the vacuolar ATPase, as well as the microsomal epoxide hydrolase [31], are two proteins that exhibit dual topologies during their initial assembly in the endoplasmic reticulum. Each form of these proteins moves to different organelles where they stably exhibit different topologies and functions. Similarly, the change in membrane topology of PLSCR1 could be related to the change in the phospholipid distribution between the two leaflets of the plasma membrane observed during monocyte-to-macrophage differentiation [25]. In addition, our findings suggest that the topological organization and membrane association of PLSCR1 is not static, but rather is a dynamic process that may vary depending on the cellular environment. For example, in keratinocytes, a significant part of the PLSCR1 protein has recently been reported to be secreted by an unconventional secretory pathway to interact with components of the dermal epidermal junction zone [32]. Interestingly, the lipid nature and properties of the plasma membrane lipid have been shown to influence PLSCR1 insertion in membranes [25]. Thus, the modification of PLSCR1 topology during monocyte-to-macrophage differentiation could be the consequence of modified membrane properties caused by the disrupted phospholipid asymmetry.

This change in membrane topology of PLSCR1 during monocyte-to-macrophage differentiation may affect the biological functions of the protein, in particular cell signaling processes. The externalization of the cytoplasmic N-terminal domain will prevent PLSCR1 from interacting directly with signal transduction proteins, and thereby could alter intracellular signaling in macrophages compared with monocytes. In turn, this change in membrane topology may also lead to the acquisition of new functions through interactions of PLSCR1 with extracellular components, such as the extracellular matrix protein 1 (ECM1) [32]. In agreement with cell surface exposure of N-terminal PLSCR1 determinants, the E1 and E2 envelope glycoproteins of the hepatitis C virus have been reported to interact directly with the region between aa 99 and 290 of PLSCR1, leading to efficient viral attachment and facilitating virus entry into hepatocytes [33].

Our data obtained in PLSCR1-depleted monocytic THP-1 cells indicate that PLSCR1 expression induced during differentiation is not required for the constitutive cell surface exposure of PS observed in macrophages. This observation is in agreement with previous studies which have recently challenged the role of PLSCR1 in regulating phospholipid movements within the plasma membrane in several experimental systems (for reviews, [2,9]). For example, ectopic overexpression of PLSCR1 in cultured mammalian cells does not correlate with cell surface exposure of PS, and platelets from PLSCR1 knockout mice have no haemostatic defects and expose PS normally when activated [34]. Finally, TMEM16F, a protein with eight transmembrane segments, has recently been proposed as the main cellular factor involved in Ca^2+^-dependent scrambling of phospholipids in the plasma membrane [35].

Interestingly, our functional analyses revealed a specific role for PLSCR1 in regulating phagocytosis of differentiated macrophages. We show that depletion of endogenous PLSCR1 stimulated FcR-mediated phagocytosis, both in shRNA-treated THP-1 cells and in bone marrow-derived macrophages from PLSCR1 knockout mice. Reciprocally, phagocytosis is inhibited in differentiated THP-1 cells overexpressing PLSCR1. Finally, immunofluorescence data demonstrated that PLSCR1 is indeed recruited both at the phagocytic cup together with F-actin, and remains associated with the phagosome during maturation, even after actin is released. In agreement with this observation, PLSCR1 has been identified in phagosomes in a large proteomic analysis of the membrane fraction of phagosomes isolated from macrophages [36].

Interestingly, PLSCR1 expression is upregulated in primary monocytes issued from systemic erythematosus lupus (SLE) patients [37], and both monocytes and macrophages from these patients, show a defect in phagocytosis [38]. This correlation between elevated PLSCR1 expression and impaired phagocytosis in monocytes and macrophages from SLE patients is consistent with our results showing that knocking-down PLSCR1 expression enhances FcR-mediated phagocytosis in macrophages, whereas overexpressing PLSCR1 inhibits this process. While these results highlight PLSCR1 as a negative modulator of phagocytosis in differentiated macrophages, further investigation is needed to explore the molecular mechanisms by which PLSCR1 regulates the phagocytic process in macrophages both in physiological and pathological situations. Interestingly, it was previously reported that FcR-mediated phagocytosis was similarly increased in PKCδ-depleted macrophages [39]. Since PKCδ has been reported to mediate both the induction of PLSCR1 gene expression in myeloid cells [27] and the phosphorylation PLSCR1 [40], we can speculate that PLSCR1 could be, at least in part, involved in the negative regulatory activity of PKCδ on the phagocytosis activity of macrophages. Another attractive line of investigation concerns the capacity of PLSCR1 to interact directly, through its proline-rich N-terminal domain, with tyrosine kinases, such as c-Abl, Lyn and Syk, which are involved in regulating FcR-mediated phagocytosis in macrophages [15,16,41].

## Conclusions

In conclusion, the results reported in the present study demonstrate that PLSCR1 expression is induced during differentiation of monocytes in macrophages and is correlated with an apparent change in the membrane topology of the protein at the cell surface of differentiated macrophages. While PLSCR1 is recruited to the phagocytic cup, as well as closed phagosomes, our results implicate PLSCR1 as a negative regulator of macrophage phagocytosis. Therefore, PLSCR1 can be added to the growing list of membrane proteins involved in the negative regulation of phagocyte functions in differentiated macrophages (for review, [42]).
